# Spatial analysis of maternal mortality in Pernambuco before and during the COVID-19 pandemic

**DOI:** 10.1590/0034-7167-2024-0416

**Published:** 2025-10-27

**Authors:** Henry Johnson Passos de Oliveira, Cristine Vieira do Bonfim, Amanda Priscila de Santana Cabral Silva

**Affiliations:** I Instituto Aggue Magalhães – FIOCRUZ. Recife, Pernambuco, Brazil; II Fundação Joaquim Nabuco. Recife, Pernambuco, Brazil; III Universidade Federal de Pernambuco. Vitória de Santo Antão, Pernambuco, Brazil

**Keywords:** Maternal Mortality, Maternal Health, SARS-CoV-2, Spatial Analysis, Ecological Studies, Mortalidad Materna, Salud Pública, COVID-19, Análisis Espacial, Estudios Ecológicos

## Abstract

**Objectives:**

to analyze the epidemiological characteristics and spatial distribution of maternal deaths in Pernambuco before (2017-2019) and during (2020-2022) the pandemic.

**Methods:**

ecological study of spatial analysis. Using data from the Mortality and Live Birth Information Systems, maternal mortality ratios by municipality were calculated and smoothed using the local empirical Bayesian method, and spatial autocorrelation was assessed using the Moran index.

**Results:**

467 maternal deaths have been reported, with a higher incidence during the COVID-19 pandemic, constituting the main cause of maternal death. The spatial analysis identified priority areas in both periods, with the concentration of risk areas remaining in the state’s Agreste region.

**Conclusions:**

identifying priority areas is crucial as it allows for effective local action and, with appropriate policies and investments, it is possible to reduce maternal deaths and achieve the targets of the Sustainable Development Goals.

## RESUMEN

**Objetivos:**

analizar las características epidemiológicas y la distribución espacial de las muertes maternas en Pernambuco, Brasil, entre 2017 y 2022.

**Métodos:**

estudio ecológico cuyas unidades de análisis espacial fueron los municipios. Se calculó la razón de mortalidad materna por municipio y, mediante el índice de Moran, se aplicó la técnica de autocorrelación espacial.

**Resultados:**

de las 467 muertes maternas registradas, 242 (51,8%) ocurrieron durante la pandemia (2020-2022). La COVID-19 fue la principal causa (n = 50). Las mujeres con menos de ocho años de educación y las madres primerizas tenían 40% y 46% más de probabilidades de morir, respectivamente. Entre quienes no asistieron a las consultas prenatales, el riesgo fue 82% mayor. El análisis espacial identificó áreas prioritarias en ambos períodos. Estas áreas se concentraron, en su mayor parte, en la región Agreste del estado.

**Conclusiones:**

la mayor concentración de áreas prioritarias en la región Agreste de Pernambuco refuerza las dificultades de acceso y atención a los servicios de salud.

## INTRODUCTION

Maternal death is an unacceptable event, which is directly related to living conditions, access to and quality of health services, in addition to being a public health problem worldwide and in Brazil(1,2,3,4). The maternal mortality ratio (MMR) is the indicator that expresses the risk of a woman dying from obstetric causes(2). This indicator is an excellent parameter of quality and access to health services(3). With sensitivity to the living conditions of a given place(4).

The commitment to reduce deaths among pregnant and postpartum women was ratified in 2015 through the new global agenda of the Sustainable Development Goals (SDGs)(5). For Brazil, the proposed goal was to reduce maternal mortality to 30 deaths per 100,000 LB by the year 2030. In addition to ensuring universal access to sexual and reproductive health services, including reproductive planning(5,6).

Despite ongoing global efforts to improve reproductive and maternal health care, the number of maternal deaths remains high(7). The spatial distribution of maternal deaths reflects social vulnerabilities in geographic spaces, producing patterns that show a concentration of the risk of death in the poorest areas(8). The lack of structure in low-complexity health services and limited resources increase the risk of maternal death(9). Events that cause health crises, such as the COVID-19 pandemic, reinforce the weaknesses of health systems, increasing gaps in access and health care(10,11).

A study conducted in Brazil using data from the acute respiratory syndrome surveillance system, collected up to July 2020, identified 204 maternal deaths from COVID-19. Of these, 5.9% were not hospitalized, 39.7% did not have access to the intensive care unit, 42.6% did not receive mechanical ventilation, and 25.5% did not even use any ventilatory support. Physical barriers are attributed as a consequence , such as distance to the reference service, and/or structural, such as the unavailability of the service due to overcrowding(12). In countries with high fertility rates and limited availability, quality and access to health resources, the risk of maternal death from COVID-19 is even greater(13).

In Brazil, between March 2020 and June 2021, an ecological study used spatial analysis to assess, among other things, the behavior of the maternal mortality ratio (MMR) due to COVID-19 according to socioeconomic characteristics and the availability of health resources(9). The results of the study revealed that there was an increase in MMR with the increase in the social vulnerability index, the Gini index and unemployment(9).

It is important to identify priority areas in order to guide planning aimed at an equitable distribution of health services. The study seeks to contribute to the understanding of how the COVID-19 pandemic affected the spatial distribution of maternal mortality, in addition to identifying priority areas with disparities in the distribution of the risk of death due to obstetric causes. In this way, intersectoral planning and rational allocation of resources in priority areas with a focus on reducing maternal mortality are possible(14).

## OBJECTIVES

To analyze the epidemiological characteristics and spatial distribution of maternal deaths in Pernambuco, Brazil, in the pre-pandemic (2017-2019) and pandemic (2020-2022) triennium.

## METHODS

### Ethical aspects

The research was conducted in accordance with the guidelines of the Declaration of Helsinki and the Resolution of the National Health Council No. 466 of 2012, which deals with the ethical aspects for studies involving human beings. The research had a letter of consent from the State Department of Health of Pernambuco and was approved by the Ethics Committee for Research involving Human Beings of the Aggeu Magalhães Institute.

### Study design, period and location

This is an ecological study with a spatial approach, with municipalities in the state of Pernambuco as the unit of analysis, between the pre-pandemic (2017-2019) and pandemic (2020-2022) trienniums. For the year 2022, the data is provisional and subject to change.

### Population: inclusion and exclusion criteria

The study was made of maternal deaths of residents in Pernambuco. As a definition for maternal death, the death of a woman during the pregnancy-puerperal period, up to 42 days after the end of pregnancy, regardless of the location or duration of the pregnancy, was considered due to any cause developed or aggravated during the pregnancy and puerperal period, directly or indirectly, with the exception of accidental and incidental causes(15).

Data from the Mortality Information System, and data from the Live Birth Information System (Sinasc) were used to calculate the MMR. The MMR were categorized as low (MMR < 20 per 100,000 Live Births – LB); medium (MMR 20 – 50/100,000 LB); high (MMR 50 to 150 per 100,000 LB); or very high (> 150 per 100,000 LB)(16). The data were made available by the State Health Department of Pernambuco on December 4, 2023. For spatial analysis, the cartographic grids were obtained from the Brazilian Institute of Geography and Statistics (IBGE), available on the website www.ibge.gov.br, accessed on December 16, 2023.

As inclusion criteria, deaths from Chapter XV (Pregnancy, childbirth and puerperium – O00 to O99) of the International Statistical Classification of Diseases and Related Health Problems (ICD-10) were included. Deaths from obstetric tetanus (A34), placental neoplasia (D39.2), hypopituitarism (E23.0), mental and behavioral disorders associated with the puerperium (F53) and puerperal osteomalacia (M83.0), as they were deaths from obstetric causes classified outside Chapter XV, were included(17).

Deaths after 42 days to less than one year after the end of pregnancy (O96) and deaths due to sequelae occurring more than one year after delivery (O97) were excluded, as they were not included in the MMR. Codes O08 (complications resulting from abortion and molar ectopic pregnancy), O30 (multiple pregnancy) and O80 to O84 (Childbirth) were excluded as they were not the underlying cause of death(17).

Fernando de Noronha was not considered for spatial analysis as it does not contain territorial continuity.

### Study protocol

Variables related to the year and municipality of residence were used to calculate the MMR grouped by three-year period (2017-2019 and 2020-2022). The demographic variables used were age group (<35 years; ≥35 years), race/skin color (black – black and brown –, non-black) and education level (<8; ≥8 years of education). The obstetric history used was previous pregnancy (primiparous or multiparous), history of childbirth (transvaginal or cesarean) and history of abortion (yes or no). In the case of variables related to pregnancy, whether any prenatal consultation was attended (yes or no) and the number of prenatal consultations (<6 or ≥6 consultations).

Finally, the variables related to the conditions and causes of death: place of occurrence (hospital, other health facilities, home, public road or others), pregnancy-puerperal period (pregnancy or puerperium), performance of autopsy (yes or no).The causes of death were classified as direct and indirect obstetric and their subgroups used to present the table of causes of death in the present study.

Direct obstetric causes refer to deaths resulting from complications during pregnancy, childbirth or the puerperium. These deaths may be attributed to inadequate interventions, omissions, incorrect treatment, or a chain of events associated with these complications, such as hypertension developed during the pregnancy-puerperal period, embolism, hemorrhage, puerperal infection, abortion, pregnancy ending in abortion, other complications related to labor and delivery, genitourinary tract infections during pregnancy, cardiomyopathy in the puerperium, among other direct obstetric causes(17).

Indirect obstetric causes are those in which death results from a pre-existing disease in the mother or developed during pregnancy, which is not directly due to obstetric causes but is aggravated by the physiological effects of pregnancy. These include diseases of the circulatory system (DCS), specified diseases and conditions (SDA), diseases of the respiratory system (RSD), diseases of the digestive system (DSD), pre-existing hypertension, mental disorders, other blood diseases, endocrine diseases and infectious-parasitic diseases that complicate pregnancy, childbirth and the puerperium(17).

Deaths from COVID-19 were presented disaggregated from infectious and parasitic diseases complicating pregnancy, childbirth and the puerperium.

### Analysis of results and statistics

We sought to describe the epidemiological profile and clinical characteristics of maternal deaths in the pre-pandemic (2017-2019) and pandemic (2020-2022) three-year periods, through absolute and relative frequencies, as well as to perform spatial analysis for these periods.

The MMRs of the study periods were smoothed using the local empirical Bayesian method. This adjustment sought to minimize the effects of random fluctuations resulting from small numbers of maternal deaths in certain municipalities. The global Moran index was used to verify the spatial autocorrelation of the ratios. This index ranges from -1 to +1, where zero indicates no autocorrelation and values closer to the extremes indicate positive or negative autocorrelation.

The Box Map and Moran Map of maternal mortality ratios were constructed. The clusters were categorized into four quadrants: Q1, Q2, Q3 and Q4. The points located in Q1 and Q2 indicate territories with similarity between their neighbors. Q1 (positive value and positive mean) and Q2 (negative value and negative mean). The points located in Q3 and Q4 indicate territories that are not similar to their neighbors. In this case, Q3 (negative value and positive mean) and Q4 (positive value and negative mean). The areas located in Q3 and Q4 can be classified as transition areas, since they do not follow the pattern observed for their neighbors(18).

The results were spatialized in the Moran scatter diagram called Box Map and Moran Map. In the Box Map, the clusters are presented regardless of statistical significance; and in the Moran Map, the clusters with significance (p-value < 0.05) are highlighted. High-risk areas corresponded to those in which the municipality had a high mortality rate and its neighbors also had high rates, classified in Q1.

TerraView (version 4.2) was used to calculate and analyze spatial autocorrelation indicators. This program calculates the neighborhood matrix (proximity) automatically, taking into account the polygons, in the case of the study the contiguous municipalities. The results were presented graphically using the QGIS program (version 2.18).

## RESULTS

**Figure 1:**
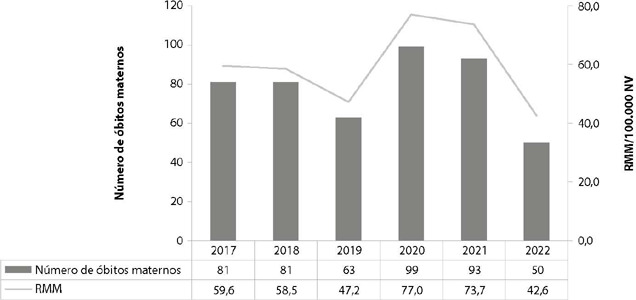
Graph of the number of maternal deaths and maternal mortality ratio in Pernambuco, Brazil, 2017 to 2022

During the study period, 467 maternal deaths were recorded in SIM. Of these, 242 (51.8%) occurred during the pandemic years. The year 2020 presented a MMR of 77 per 100,000 LB, which represents an increase of 63.1% compared to 2019, which presented a MMR of 47.2 per 100,000 LB (Figure 1).

In both study periods, approximately 77% of maternal deaths were among black women. More than 45% had more than eight years of schooling (n = 216; 46.3%) and 68% were between 10 and 34 years of age (n = 319; 68.3%). Most were multigravidas (n = 329; 70.4%) with a history of transvaginal delivery (n = 195; 59.3%) and had attended prenatal care (n = 373; 79.9%) with six or more consultations (n = 205; 55%). In general, deaths occurred in a hospital setting (n = 405; 86.7%), in the puerperium (n = 319; 68.3%) and no autopsy was performed (n = 304; 65.1%) (Table 1).

**Table 1:** Number of maternal deaths and proportion by demographic variable, obstetric history, variables related to pregnancy and care, Pernambuco, Brazil, 2017-2019 (pre-pandemic) and 2020-2022 (pandemic)*

Variables	Pre-pandemic		Pandemic	
	(n)	(%)	(n)	(%)
Sociodemographic variables				
Race/skin color				
Black	173	76.9	187	77.3
Non-black	45	20.0	51	21.1
Ignored	7	3.1	4	1.66
Education				
< Eight years of study	99	44.0	104	43.0
≥ Eight years of study	102	45.3	114	47.1
Ignored	24	10.7	24	9.9
Age range				
10 to 34 years old	155	68.9	164	67.8
35 to 49 years old	70	31.1	78	32.2
Obstetric history				
Gestation				
Primigravida	55	24.4	69	28.5
Multigesta	167	74.2	162	66.9
Ignored	3	1.3	11	4.6
Birth history				
Transvaginal	99	59.3	96	59.3
Caesarean section	81	48.5	80	49.4
Ignored	3	1.8	11	6.8
Variables related to pregnancy				
Completed prenatal care				
Yes	181	80.4	192	79.3
No	36	16.0	36	14.9
Ignored	8	3.6	14	5.8
Number of prenatal consultations				
< six consultations	89	49.2	79	41.2
≥ six or more consultations	92	50.8	113	58.8
Place of occurrence				
Hospital	188	83.6	217	89.7
Other health facilities	15	6.7	16	6.6
Home	11	4.9	8	3.3
Public road	2	0.9	1	0.4
Others	9	4.0	-	-
Time of death (d)				
During pregnancy	49	21.8	39	16.1
During labor	12	5.3	7	2.9
During miscarriage	8	3.6	10	4.1
In the puerperium (up to 42 days after birth)	146	64.9	173	71.5
Ignored	10	4.4	13	5.4
Performed autopsy (e)				
Yes	98	43.6	26	10.7
No	109	48.4	195	80.6
Ignored	18	8.0	21	8.7
Total	225	100	242	100

*The pre-pandemic and pandemic periods correspond, respectively, to the periods before and during the COVID-19 pandemic; Data collected on December 4, 2023. Source: SIM/GVEV/DGIE/SEVSAP/SES-PE.

Regarding the causes of maternal death, in the period before the pandemic, hypertension (n = 39; 26.5%) and hemorrhages (n = 38; 25.9%) were the main groups of direct obstetric causes. As an indirect cause of maternal death, diseases of the circulatory system represented the largest proportions (n = 22; 30.6%). During the pandemic period, the main cause of death was COVID-19 (n = 50; 46.7%) (Table 2).

**Table 2:** Groups of causes of maternal deaths of residents in Pernambuco, Brazil, 2017-2019 (pre-pandemic) and 2020-2022 (pandemic)*

Maternal death cause group	Pre-pandemic		Pandemic	
	(n)	(%)	(n)	(%)
Direct maternal obstetric causes				
Direct hypertensions	39	26.5	49	36.8
Bleeding (including uterine inertia)	38	25.9	20	15.0
Puerperal infection	8	5.4	15	11.3
Embolisms	16	10.9	17	12.8
Ectopic pregnancy	5	3.4	7	5.3
Pregnancy ending in miscarriage	8	5.4	3	2.3
Hydatidiform mole	2	1.4	-	-
Other direct obstetric causes	31	21.1	22	16.5
Subtotal	147	65.3	133	55.0
Indirect maternal obstetric causes				
DCS complicating pregnancy, childbirth or puerperium	22	30.6	12	11.2
COVID-19	-	-	50	46.7
Other viral diseases complicating pregnancy, childbirth or puerperium	1	2.8	3	2.8
Other DCSs complicating pregnancy, childbirth or puerperium	12	16.7	5	4.7
RSD complicating pregnancy, childbirth or puerperium	11	15.3	8	7.5
DSD complicating pregnancy, childbirth or puerperium	2	2.8	4	3.7
HIV/Aids^1^	6	6.9	2	1.9
Other indirect obstetric causes	18	25.0	23	21.5
Subtotal	72	32.0	107	44.2
Obstetric death from unspecified cause	6	2.7	2	0.8
Total maternal deaths (up to 42 days)	225	100.0	242	100.0

*The pre-pandemic and pandemic periods correspond, respectively, to the periods before and during the COVID-19 pandemic; DCS – diseases of the circulatory system; SDA – specified diseases and conditions; RSD – diseases of the respiratory system; DSD – diseases of the digestive system. Source: SIM/GVEV/DGIE/SEVSAP/SES-PE.

**Figure 2:**
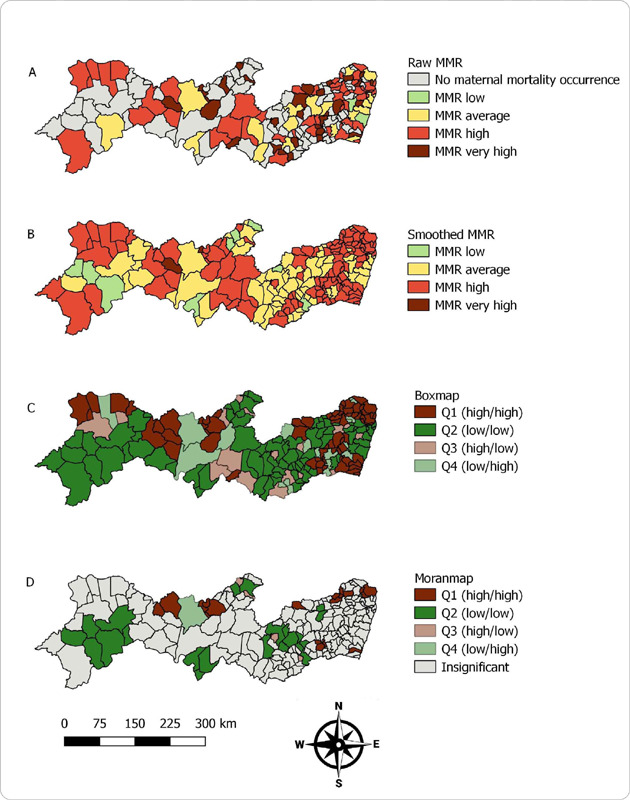
(A) Thematic map of maternal mortality ratio; (B) Thematic map of smoothed maternal mortality ratio; (C) Box Map of maternal mortality ratio; (D) Moran Map of maternal mortality ratio. Pernambuco, Brazil, 2017–2019 (pre-pandemic)

*Source: SIM/GVEV/DGIE/SEVSAP/SES-PE.*

In the spatial distribution of MMR by municipalities, 78 (42.2%) presented a high or very high MMR. Mirandiba, located in the backlands of the state, presented the highest MMR (393.2 per 100,000 LB) (Figure 2A). When smoothing the MMR, an increase in the number of municipalities (n = 110; 59.8%) with high or very high MMR was observed (Figure 2B). The global Moran index (I) was 0.407 (p = 0.001), indicating the existence of spatial dependence. In the Box Map (Q1 – high/high), seven clusters were located distributed across all mesoregions of the state (Figure 2C). The Moran Map identified five clusters formed by 17 priority municipalities and two isolated municipalities. The main cluster is formed by five municipalities located in the Agreste (4) and Mata (1) regions of Pernambuco (Figure 2D).

*Data collected on December 4, 2023*

**Figure 3:**
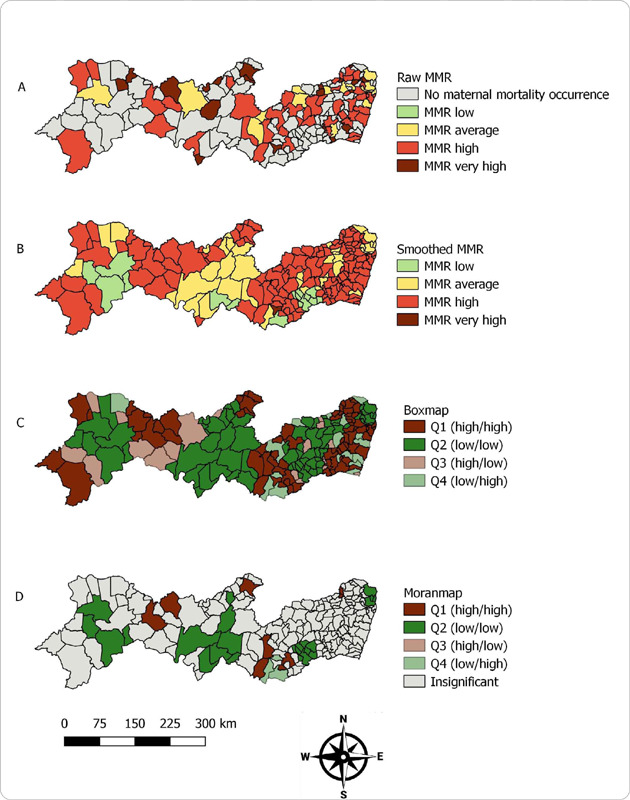
(A) Thematic map of maternal mortality ratio; (B) Thematic map of smoothed maternal mortality ratio; (C) Box Map of maternal mortality ratio; (D) Moran Map of maternal mortality ratio. Pernambuco, Brazil, 2020–2022 (pandemic)

*Source: SIM/GVEV/DGIE/SEVSAP/SES-PE,*

During the pandemic period, 66 (35.7%) municipalities had a high or very high MMR. The municipality with the highest ratio (720.7 per 100,000 live births) was Saloá, located in the Agreste region of the state (Figure 3A). After the Bayesian local empirical analysis, there was an increase in the number of municipalities with high and very high MMR (n = 130; 70.7%) in all mesoregions of the state (Figure 3B). The global Moran’s I index indicated positive spatial autocorrelation (0.313; P = 0.002). In the Box Map (Q1 – high/high), seven clusters were located distributed in 76 (41.3%) municipalities in all mesoregions (Figure 3C). The Moran Map identified three clusters formed by two priority municipalities each, all in the Agreste region of the state, in addition to three isolated municipalities in the backlands region (Figure 3D).

*Data collected on December 4, 2023*

## DISCUSSION

During the study period, most deaths occurred during the pandemic, with 2020 recording the highest MMR in the historical series. There was a predominance of deaths among black women, with more than eight years of education and ages between 10 and 34 years. In general, they were multiparous with a history of transvaginal delivery. Most had at least six prenatal visits. Deaths were concentrated in the postpartum period and occurred predominantly in hospital services. The main cause of death, which was previously hypertension and hemorrhage, became COVID-19. For the spatial analysis, in the period before the pandemic, 19 priority municipalities were observed. Of these, 17 formed five clusters and two were isolated. During the pandemic, there was a reduction in this number, with the Moran Map identifying nine priority municipalities. However, the main priority areas for intervention, in the pre-pandemic and pandemic periods, are concentrated in the state’s Agreste region.

In this study, there was an increase in the MMR in Pernambuco. The exploratory research conducted in Brazil aimed to analyze the effects of the COVID-19 pandemic on maternal mortality. As a result, excess mortality during the pandemic was identified, being related to a multicausality with articulation beyond biological aspects, involving political aspects, such as the functioning and structure of the Unified Health System and its capacity to manage the health crisis(19). The pandemic has introduced additional barriers to obstetric health care, which directly affects maternal mortality. The occurrence of death from obstetric causes is influenced by the quality of care, which includes access to services, the availability of essential resources and the adequacy of health practices during the pregnancy-puerperal period(20). The disruption of health services caused by the pandemic has also affected primary care services, resulting in a reduction in prenatal consultations(21). Therefore, diseases such as pre-eclampsia/eclampsia, urinary infections, hemorrhages, among others that should be diagnosed and treated prenatally, evolved with unfavorable outcomes(22). In this way, priority must be given to recovering the health system in Brazilian territories, especially in the post-pandemic scenario(23). In Pernambuco, it is necessary to identify priority territories and act urgently on them to minimize the impacts of the pandemic and resume the project to reduce maternal mortality.

In the spatial analysis, in both periods, the identification of priority municipalities has been observed. These areas have a higher risk of maternal mortality. Similarly, a study conducted in the state of Alagoas with data on deaths from obstetric causes between 1996 and 2016, after spatial analysis using the Moran Index, identified regions with a higher risk of maternal death(24). In this way, the identification of priority areas can assist in health planning, monitoring and equitable targeting of health interventions and resources that are sometimes insufficient and scarce(25).

The clusters identified in the research are primarily formed by municipalities in the state’s Agreste region. According to data from the Municipal Human Development Index (MHDI), approximately 77.5% of the municipalities in the Agreste region of Pernambuco have an MHDI classified as low(26). The MHDI uses data on health, education and income per person. This makes it possible to measure the level of development of the population in different regions(27). A study conducted in Pernambuco between 2010 and 2020 assessed the bivariate spatial autocorrelation between maternal mortality and MHDI using Moran’s spatial statistics. As a result, it was found that the municipalities with the highest risk of maternal death had low human development and little structural social capital(28).

According to data from the Monthly Monitoring of the Estimated Rate of the Pernambuco Population in a Situation of Extreme Poverty entered in the Single Registry in relation to the population estimated by the IBGE, Pernambuco had, in August 2023, approximately 37.8% of the population in a situation of extreme poverty. In Agreste, 95.8% of the municipalities have percentages higher than the state average and approximately 67.6% of the municipalities have more than 50% of their resident population in a situation of extreme poverty(26). Financial barriers can negatively affect maternal health in different regions, as they promote, among other things, difficulties in accessing regular and emergency medical care(29).

A study conducted in Brazil, with data on records of 23,823 cases of COVID-19 and 1,991 deaths among pregnant and postpartum women, used the bivariate Moran to assess the spatial correlation of cases and deaths of pregnant and postpartum women due to COVID-19 with socioeconomic and social vulnerability data. As a result, smaller municipalities and those with a higher degree of vulnerability had the highest maternal mortality ratios(30).

Commonly, health services in the interior are not adequately prepared to provide the necessary care to pregnant and postpartum women. This may include a lack of qualified human resources, outdated structures and low occupancy rates, which compromise the quality of care. Due to the ineffectiveness of local care provision, pregnant women often need to be transferred to better equipped health facilities(31). Transportation difficulties as well as the distance to reference services also represent a challenge(32). Regions with greater inequality in the quality and quantity of health services increase negative obstetric outcomes(33).

An exploratory study conducted in Brazil aimed to analyze the process of regionalization in health in Brazil from the perspective of municipal managers. As a result, it was observed that access to health services, especially those with greater technological resources, is difficult and centralized, which requires users to travel long distances to obtain care(34). Another study conducted in the Agreste region of the state of Pernambuco, with the aim of evaluating access to health services, used geographic data relating to the distance traveled and the travel time between the municipality of residence and the birth. As a result, it was observed that 17.1% of women who had their birth classified as normal risk and 49.1% classified as high risk needed to travel more than 120 km to the reference health service(35).

Structural problems, such as failures in the regionalization and decentralization of health services, as well as in regulatory systems, negatively impact the risk of unfavorable obstetric outcomes. Therefore, the organization of health care networks in the territory, fully implemented regulatory systems and qualified regional planning are decisive in facilitating or hindering access(31,32).

The combination of these factors highlights the complexity of the challenges faced by health systems in peripheral areas, especially in contexts where health resources are insufficient and inadequate to deal with obstetric emergencies. This may contribute to the increase in maternal mortality in these regions.

This research points to a higher number of maternal deaths during the pandemic. If not addressed as a priority, this scenario could result in greater difficulties in reducing maternal mortality in Pernambuco. Brazil’s commitment to reduce mortality due to obstetric causes to 30 maternal deaths per 100,000 live births will become yet another commitment to be postponed.Therefore, there is an urgent need to understand the direct and indirect effects of the COVID-19 pandemic in the short and long term on obstetric outcomes.

Achieving the target proposed by the SDG represents a challenge that must be a management priority. It is important to ensure the continuity of obstetric services beyond the pandemic as a fundamental strategy for restructuring and mitigating the negative impacts on maternal and child health. This includes public policies that ensure the continuity of prenatal care and other essential care, especially for vulnerable areas. Another important factor is the role of health managers in decentralizing and qualifying health services in peripheral areas. These measures translate into an opportunity to reduce preventable deaths during pregnancy, childbirth and the postpartum period.

### Study limitations

As a limitation of the study, since it is an analysis using secondary data, the inadequate completion of death certificates, specifically regarding the causes of death and the time of death during the pregnancy-puerperal period, produces under-reporting of maternal deaths. Errors in coding and/or errors in typing the death certificate into the system contribute to underreporting and affect the estimates of the indicator. Added to this is the matter that. the data for 2022 is preliminary. However, the data from the Health Information Systems in Pernambuco have shown satisfactory quality related to vital events and reaffirm the use of these systems as reliable instruments for analyzing the health situation(36). Furthermore, since this is an ecological study, it is vulnerable to the ecological fallacy. Therefore, its results cannot be inferred at an individual level. Since it is a descriptive study, it does not have the capacity to infer a cause-effect relationship.

### Contributions to the areas of Nursing, Health and Public Policies

Nursing is essential for the performance of primary health care services, such as prenatal consultations. This level of care has the capacity to resolve approximately 80% of health problems. Maternal deaths occur, in most cases, due to lack of access and assistance to basic and regular health actions and services. The results of the study highlight priority areas for intervention that may be of interest to public management, both for the requalification and decentralization of health services in the state.

## CONCLUSIONS

Maternal deaths were more frequent during the pandemic period, with changes in priority areas between periods. Despite this, the priority areas, both in the period before the pandemic and during the pandemic period, are concentrated in the Agreste region of Pernambuco.

The geographic identification of priority areas for maternal deaths represents an opportunity for local action. It is possible, with policies and investments in health, to reduce the occurrence of maternal deaths in regions of greater risk. With this, it is possible for Pernambuco, with prioritization, focus and commitment, to reach the goal established by the SDGs.

## Data Availability

The research data are available only upon request.
