# Supplementary material for: Spatial analysis of maternal mortality in Pernambuco before and during the COVID-19 pandemic
Source: Rev Bras Enferm. 2025 Oct 27;78(Suppl 1):e20240416. doi: 10.1590/0034-7167-2024-0416 (PMC12563838; doi:10.1590/0034-7167-2024-0416)
Supplement: Translation

# Translation: Análise espacial da mortalidade materna antes e durante a pandemia pela COVID-19

**DOI:** 10.1590/0034-7167-2024-0416pt

**Authors:** Henry Johnson Passos de Oliveira, Cristine Vieira do Bonfim, Amanda Priscila de Santana Cabral Silva

**Affiliations:** I Instituto Aggue Magalhães – FIOCRUZ. Recife, Pernambuco, Brasil; II Fundação Joaquim Nabuco. Recife, Pernambuco, Brasil; III Universidade Federal de Pernambuco. Vitória de Santo Antão, Pernambuco, Brasil

**Keywords:** Mortalidade Materna, Saúde Pública, COVID-19, Análise Espacial, Estudos Ecológicos

## Abstract

**Objetivos:**

analisar as características epidemiológicas e a distribuição espacial dos óbitos maternos em Pernambuco, Brasil, entre 2017 e 2022.

**Métodos:**

estudo ecológico cujas unidades de análise espacial foram os municípios. Calculou-se a razão de mortalidade materna por município e, por meio do índice de Moran, aplicou-se a técnica de autocorrelação espacial.

**Resultados:**

dos 467 óbitos maternos registrados, 242 (51,8%) ocorreram durante a pandemia (2020-2022). A COVID-19 foi a principal causa (n = 50). As mulheres com menos de oito anos de estudo e as primigestas apresentaram 40% e 46% mais chance de óbito, respectivamente. Entre as que não realizaram consultas de pré-natal, o risco foi 82% maior. A análise espacial identificou áreas prioritárias em ambos os períodos. Essas áreas se concentraram, em sua maior parte, no Agreste do estado.

**Conclusões:**

a maior concentração de áreas prioritárias no Agreste pernambucano reforça as dificuldades de acesso e assistência aos serviços de saúde.

## INTRODUÇÃO

O óbito materno é um evento inaceitável, que possui relação direta com as condições de vida, o acesso e a qualidade dos serviços de saúde, além de ser um problema de saúde pública no mundo e no Brasil(1,2,3,4). A razão de mortalidade materna (RMM) é o indicador que expressa o risco de uma mulher morrer por causas obstétricas(2). Esse indicador se configura como um excelente parâmetro de qualidade e de acesso aos serviços de saúde(3). Com sensibilidade às condições de vida de um determinado lugar(4).

O compromisso em reduzir os óbitos de gestantes e puérperas foi ratificado em 2015 por meio da nova agenda global dos Objetivos do Desenvolvimento Sustentável (ODS)(5). Para o Brasil, foi proposto como meta reduzir a mortalidade materna para 30 óbitos por 100.000 NV até o ano de 2030. Além de garantir o acesso universal aos serviços de saúde sexual e reprodutiva, incluindo o planejamento reprodutivo(5,6).

Apesar dos esforços mundiais contínuos voltados para a melhoria da atenção à saúde reprodutiva e materna, o número de óbitos maternos ainda é alto(7). A distribuição espacial dos óbitos maternos reflete vulnerabilidades sociais nos espaços geográficos, produzindo padrões que apresentam uma concentração do risco de morrer nas áreas mais pobres(8). A falta de estrutura dos serviços de saúde de baixa complexidade e os recursos limitados potencializam o risco de morte materna(9). Eventos que ocasionam crises sanitárias, como a pandemia pela COVID-19, reforçam as fragilidades dos sistemas de saúde, potencializando as falhas no acesso e na assistência à saúde(10,11).

Uma pesquisa realizada no Brasil com dados do sistema de vigilância de síndrome respiratória aguda, coletados até julho de 2020, identificou 204 mortes maternas por COVID-19. Destas, 5,9% não foram internadas, 39,7% não tiveram acesso à unidade de terapia intensiva, 42,6% não receberam ventilação mecânica, e 25,5% sequer utilizaram algum suporte ventilatório. Atribui-se como consequência as barreiras físicas, como a distância até o serviço de referência, e/ou estruturais, como a indisponibilidade do serviço por superlotação(12). Em países que possuam taxas de fecundidade elevadas e limitação na disponibilidade, na qualidade e no acesso aos recursos em saúde, revela-se ainda mais o risco de morte materna pela COVID-19(13).

No Brasil, entre março de 2020 a junho de 2021, um estudo ecológico utilizou a análise espacial para avaliar, entre outros, o comportamento da razão da mortalidade materna (RMM) pela COVID-19 de acordo com as características socioeconômicas e a disponibilidade de recursos de saúde^9^. Os resultados do estudo revelaram que houve um incremento nas RMM com o aumento do índice de vulnerabilidade social, do índice de Gini e do desemprego(9).

É importante identificar áreas prioritárias a fim de orientar o planejamento voltado a uma distribuição equânime dos serviços de saúde. O estudo busca contribuir na compreensão decomo a pandemia pela COVID-19 afetou a distribuição espacial da mortalidade materna, além de identificar áreas prioritárias com disparidades na distribuição do risco de morte por causa obstétrica. Dessa forma, é possível um planejamento intersetorial e dimensionamento racional de recursos em áreas prioritárias com foco na redução da mortalidade materna(14).

## OBJETIVOS

Analisar as características epidemiológicas e a distribuição espacial dos óbitos maternos em Pernambuco, Brasil, no triênio pré-pandêmico (2017-2019) e pandêmico (2020-2022).

## MÉTODOS

### Aspectos éticos

A pesquisa foi conduzida de acordo com as diretrizes da Declaração de Helsinque e a Resolução do Conselho Nacional de Saúde nº 466 de 2012, que trata sobre os aspectos éticos para estudos envolvendo seres humanos. A pesquisa contou com a carta de anuência da Secretaria Estadual de Saúde de Pernambuco e foi aprovada pelo Comitê de Ética em Pesquisas em Seres Humanos do Instituto Aggeu Magalhães.

### Desenho, período e local do estudo

Trata-se de um estudo ecológico com abordagem espacial, tendo como unidade de análise os municípios do estado de Pernambuco, entre os triênios pré-pandêmicos (2017-2019) e pandêmicos (2020-2022). Para o ano de 2022, o dado é provisório e sujeito a alterações.

### População: critérios de inclusão e exclusão

O estudo foi composto pelos óbitos maternos de residentes em Pernambuco. Como definição para o óbito materno, considerou-se a morte de uma mulher durante o período gravídico-puerperal, até 42 dias após o término da gestação, independentemente da localização ou duração da mesmapor qualquer causa desenvolvida ou agravada no período gestacional e puerperal de forma direta ou indireta, à exceção das causas acidentais e incidentais(15).

Foram utilizados dados provenientes do Sistema de Informação sobre Mortalidade e, para cálculo da RMM, foram utilizados os dados do Sistema de Informações sobre Nascidos Vivos (Sinasc). As RMM foram categorizadas em baixa (RMM < 20 por 100.000 Nascidos Vivos – NV); média (RMM 20 – 50/100.000 NV); alta (RMM 50 A 150 por 100.000 NV); ou muito alta (> 150 por 100.000 NV) (16). Os dados foram disponibilizados pela Secretaria Estadual de Saúde de Pernambuco em 04 de dezembro de 2023. Para a análise espacial, as malhas cartográficas foram obtidas a partir do Instituto Brasileiro de Geografia e Estatística (IBGE), disponibilizadas no site www.ibge.gov.br, acessado em 16 de dezembro de 2023.

Como critério de inclusão, os óbitos do capítulo XV (Gravidez parto e puerpério – O00 a O99) da Classificação Estatística Internacional de Doenças e Problemas Relacionados à Saúde (CID-10). Os óbitos por tétano obstétrico (A34), neoplasia da placenta (D39.2), hipopituitarismo (E23.0), transtornos mentais e comportamentais associados ao puerpério (F53) e osteomalácia puerperal (M83.0), por se tratarem de óbitos por causas obstétricas classificadas fora do capítulo XV, foram incluídos(17).

Foram excluídos os óbitos após 42 dias até menos de um ano após o término da gestação (O96) e os óbitos por sequelas que ocorrem mais de um ano após o parto (O97), por não computarem para a RMM. Os códigos O08 (complicações consequentes a aborto e gravidez ectópica molar), O30 (gestação múltipla) e O80 a O84 (Parto) foram excluídos por não se tratarem de causa básica de morte(17).

Fernando de Noronha não foi considerado para a análise espacial por não conter continuidade territorial.

### Protocolo do estudo

Foram utilizadas variáveis referentes ao ano e município de residência para cálculo da RMM agrupado por triênio (2017-2019 e 2020-2022). Como variável demográfica, foi utilizada a faixa etária (< 35 anos; ≥35 anos), a raça/cor da pele (negras –pretas e pardas –, não negras) e a escolaridade (<8; ≥8 anos de estudos). Os antecedentes obstétricos utilizados foram a gestação anterior (primigesta ou multigesta), o histórico de parto (transvaginal ou cesariana) e o histórico de aborto (sim ou não). No caso das variáveis relacionadas à gestação, se realizou alguma consulta de pré-natal (sim ou não) e o número das consultas de pré-natal (< 6 ou ≥6 consultas).

Por fim, as variáveis relacionadas às condições e causas do óbito: local de ocorrência (hospital, outros estabelecimentos de saúde, domicílio, via pública ou outros), período gravídico-puerperal (gestação ou puerpério), realização de necropsia (sim ou não). As causas de morte foram classificadas em obstétrica direta e indireta e seus subgrupos utilizados para apresentar a tabela de causa de morte no presente estudo.

As causas obstétricas diretas referem-se às mortes resultantes de complicações durante a gravidez, o parto ou o puerpério. Essas mortes podem ser atribuídas a intervenções inadequadas, omissões, tratamento incorreto, ou a uma cadeia de eventos associados a essas complicações, como hipertensão desenvolvida durante o período gravídico-puerperal, embolia, hemorragia, infecção puerperal, aborto, gravidez que termina em aborto, outras complicações relacionadas ao trabalho de parto e ao parto, infecções do trato geniturinário durante a gravidez, cardiomiopatia no puerpério, entre outras causas obstétricas diretas(17).

As causas obstétricas indiretas são aquelas em que a morte resulta de uma doença pré-existente na mãe ou desenvolvida durante a gravidez, que não se deve diretamente a causas obstétricas, mas que é agravada pelos efeitos fisiológicos da gestação. Isso inclui doenças do aparelho circulatório (DAC), doenças e afecções especificadas (DAE), doenças do aparelho respiratório (DAR), doenças do aparelho digestivo (DAG), hipertensão preexistente, transtornos mentais, outras doenças do sangue, doenças endócrinas e doenças infecto-parasitárias que complicam a gravidez, o parto e o puerpério(17).

Os óbitos por COVID-19 foram apresentados desagregados das doenças infecciosas e parasitárias complicando a gravidez, o parto e o puerpério.

### Análise dos resultados e estatística

Buscou-se descrever o perfil epidemiológico e as características clínicas dos óbitos maternos nos triênios pré-pandêmico (2017-2019) e pandêmico (2020-2022), por meio das frequências absolutas e relativas, bem como realizar a análise espacial para esses períodos.

As RMM dos períodos do estudo foram suavizadas pelo método bayesiano empírico local. Esse ajuste buscou minimizar os efeitos das flutuações aleatórias em decorrência de pequenos números de óbitos maternos em determinados municípios. Foi utilizado o índice de Moran global para verificar a autocorrelação espacial das razões. Esse índice varia de -1 a +1, em que o zero indica ausência de autocorrelação e os valores mais próximos aos extremos indicam autocorrelação positiva ou negativa.

Foram construídos o Box Map e o Moran Map das razões de mortalidade materna. Os *clusters* foram categorizados em quatro quadrantes: Q1, Q2, Q3 e Q4. Os pontos localizados no Q1 e Q2 indicam territórios com semelhança entre os vizinhos. Q1 (valor positivo e média positiva) e Q2 (valor negativo e média negativa). Os pontos localizados no Q3 e Q4 indicam territórios não semelhantes aos seus vizinhos. Nesse caso, Q3 (valor negativo e média positiva) e Q4 (valor positivo e média negativa). As áreas localizadas no Q3 e Q4 podem ser classificadas como áreas de transição, uma vez que não obedecem ao padrão observado para seus vizinhos(18).

Os resultados foram espacializados no diagrama de espalhamento de Moran denominado de Box Map e Moran Map. No Box Map, os *clusters* são apresentados independentemente da significância estatística; e no Moran Map, evidenciam-se os *clusters* com significância (p-valor < 0,05). Áreas de alto risco corresponderam àquelas em que o município apresenta alta taxa de mortalidade e os seus vizinhos também têm altas taxas, classificadas no Q1.

O TerraView (versão 4.2) foi utilizado para o cálculo e a análise dos indicadores de autocorrelação espacial. Esse programa calcula a matriz de vizinhança (proximidade) automaticamente, levando em consideração os polígonos, no caso do estudo os municípios, contíguos. Os resultados foram apresentados graficamente por meio do o programa QGIS (versão 2.18).

## RESULTADOS

No período do estudo, foram registrados no SIM, 467 óbitos maternos. Destes, 242 (51,8%) ocorreram durante os anos pandêmicos. O ano de 2020 apresentou uma RMM de 77 por 100.000 NV, o que representa um incremento de 63,1% em comparação a 2019, que apresentou uma RMM de 47,2 por 100.1 NV (Figura 1).

**Figura 1:** Gráfico do número de óbitos maternos e da razão de mortalidade materna em Pernambuco, Brasil, 2017 a 2022

Em ambos os períodos do estudo, cerca de 77,0% dos óbitos maternos foram de mulheres negras. Mais de 45% possuíam mais de oito anos de estudo (n = 216; 46,3%) e 68,0% com idade entre 10 e 34 anos (n = 319; 68,3%). A maior parte eram multigestas (n = 329; 70,4%) com histórico de parto transvaginal (n = 195; 59,3%) e realizaram o pré-natal (n = 373; 79,9%) com seis ou mais consultas (n = 205; 55,0%). Em geral, os óbitos ocorreram em ambiente hospitalar (n = 405; 86,7%), no puerpério (n = 319; 68,3%) e não foi realizada a necropsia (n = 304; 65,1%) (Tabela 1).

**Tabela 1:** Número de óbitos maternos e proporção por variável demográfica, antecedente obstétrico, variáveis relacionadas à gestação e à assistência, Pernambuco, Brasil, 2017-2019 (pré-pandêmico) e 2020-2022 (pandêmico)*

Variáveis	Pré-pandêmico		Pandêmico	
	(n)	(%)	(n)	(%)
Variáveis sociodemográficas				
Raça/cor da pele				
Negras	173	76,9	187	77,3
Não negras	45	20,0	51	21,1
Ignorado	7	3,1	4	1,66
Escolaridade				
< Oito anos de estudo	99	44,0	104	43,0
≥ Oito anos de estudo	102	45,3	114	47,1
Ignorados	24	10,7	24	9,9
Faixa etária				
10 a 34 anos	155	68,9	164	67,8
35 a 49 anos	70	31,1	78	32,2
Antecedentes obstétricos				
Gestação				
Primigesta	55	24,4	69	28,5
Multigesta	167	74,2	162	66,9
Ignorado	3	1,3	11	4,6
Histórico de parto				
Transvaginal	99	59,3	96	59,3
Cesariana	81	48,5	80	49,4
Ignorados	3	1,8	11	6,8
Variáveis relacionadas à gestação				
Realizou o pré-natal				
Sim	181	80,4	192	79,3
Não	36	16,0	36	14,9
Ignorado	8	3,6	14	5,8
Número de consultas do pré-natal				
< seis consultas	89	49,2	79	41,2
≥ seis ou mais consultas	92	50,8	113	58,8
Local de ocorrência				
Hospital	188	83,6	217	89,7
Outros estabelecimentos de saúde	15	6,7	16	6,6
Domicílio	11	4,9	8	3,3
Via pública	2	0,9	1	0,4
Outros	9	4,0	-	-
Momento do óbito (d)				
Na gravidez	49	21,8	39	16,1
No parto	12	5,3	7	2,9
No aborto	8	3,6	10	4,1
No puerpério (até 42 dias após parto)	146	64,9	173	71,5
Ignorado	10	4,4	13	5,4
Realizou necropsia (e)				
Sim	98	43,6	26	10,7
Não	109	48,4	195	80,6
Ignorado	18	8,0	21	8,7
Total	225	100	242	100

*Os períodos pré-pandêmico e pandêmico correspondem, respectivamente, aos períodos antes e durante a pandemia pela COVID-19; Dados coletados em 4 de dezembro de 2023. Fonte: SIM/GVEV/DGIE/SEVSAP/SES-PE.

Com relação às causas de morte materna, no período anterior à pandemia, as hipertensões (n = 39; 26,5%) e hemorragias (n = 38; 25,9%) foram os principais grupos de causas obstétricas diretas. Como causa indireta de morte materna, as doenças do aparelho circulatório representaram as maiores proporções (n = 22; 30,6%). Durante o período pandêmico, a principal causa de morte foi a COVID-19 (n = 50; 46,7%) (Tabela 2).

**Tabela 2:** Grupos de causas dos óbitos maternos de residentes em Pernambuco, Brasil, 2017-2019 (pré-pandêmico) e 2020-2022 (pandêmico)*

Grupo de causa de óbitos maternos	Pré-pandêmico		Pandêmico	
	(n)	(%)	(n)	(%)
Causas maternas obstétricas diretas				
Hipertensões diretas	39	26,5	49	36,8
Hemorragias (incluindo inércia uterina)	38	25,9	20	15,0
Infecção puerperal	8	5,4	15	11,3
Embolias	16	10,9	17	12,8
Gravidez ectópica	5	3,4	7	5,3
Gravidez que termina em aborto	8	5,4	3	2,3
Mola hidatiforme	2	1,4	-	-
Outras causas obstétricas diretas	31	21,1	22	16,5
Subtotal	147	65,3	133	55,0
Causas maternas obstétricas indiretas				
DAC complicando gravidez, parto ou puerpério	22	30,6	12	11,2
COVID-19	-	-	50	46,7
Outras doenças virais complicando gravidez, parto ou puerpério	1	2,8	3	2,8
Outras DAE complicando gravidez, parto ou puerpério	12	16,7	5	4,7
DAR complicando gravidez, parto ou puerpério	11	15,3	8	7,5
DAD complicando gravidez, parto ou puerpério	2	2,8	4	3,7
HIV/Aids^1^	6	6,9	2	1,9
Outras causas obstétricas indiretas	18	25,0	23	21,5
Subtotal	72	32,0	107	44,2
Morte obstétrica de causa não especificada	6	2,7	2	0,8
Total de óbitos maternos (até 42 dias)	225	100,0	242	100,0

*Os períodos pré-pandêmico e pandêmico correspondem, respectivamente, aos períodos antes e durante a pandemia pela COVID-19; DAC – doenças do aparelho circulatório; DAE – doenças e afecções especificadas; DAR – doenças do aparelho respiratório; DAG – doenças do aparelho digestivo.

*Fonte: SIM/GVEV/DGIE/SEVSAP/SES-PE.*

Na distribuição espacial da RMM por municípios, 78 (42,2%) apresentaram uma RMM alta ou muito alta. Mirandiba, localizado no Sertão do estado, apresentou a maior RMM (393,2 por 100.000 NV) (Figura 2A). Ao realizar a suavização da RMM, observou-se ampliação do número de municípios (n = 110; 59,8%) com RMM alta ou muito alta (Figura 2B). O índice global (I) de Moran foi de 0,407(p=0,001), indicando a existência de dependência espacial. No *Box Map* (Q1 – alto/alto), foram localizados sete *clusters* distribuídos em todas as mesorregiões do estado (Figura 2C). O *Moran Map* identificou cinco *clusters* formados por 17 municípios prioritários e dois municípios isolados. O principal *cluster* é formado por cinco municípios localizados no Agreste (4) e Mata (1) de Pernambuco (Figura 2D).

**Figura 2:** (A) Mapa temático da razão de mortalidade materna; (B) Mapa temático da razão de mortalidade materna suavizada; (C) *Box Map* da razão de mortalidade materna; (D) *Moran Map* da razão de mortalidade materna, Pernambuco, Brasil, 2017–2019 (pré-pandêmico)

*Fonte: SIM/GVEV/DGIE/SEVSAP/SES-PE.*

*Dados coletados em 4 de dezembro de 2023*

Durante o período pandêmico, 66 (35,7%) municípios possuíam uma RMM alta ou muito alta. O município com maior razão (720,7 por 100.000 nascidos vivos) foi Saloá, localizado no Agreste do estado (Figura 3A). Após a análise empírica local bayesiana, houve ampliação no número de municípios com RMM alta e muito alta (n = 130; 70,7%) em todas as mesorregiões do estado (Figura 3B). O índice global de Moran I indicou autocorrelação espacial positiva (0,313; P=0,002). No *Box Map* (Q1 – alto/alto), foram localizados sete *clusters* distribuídos em 76 (41,3%) municípios em todas as mesorregiões (Figura 3C). O *Moran Map* identificou três *clusters* formados por dois municípios prioritários cada, todos no Agreste do estado, além de três municípios isolados no Sertão (Figura 3D).

**Figura 3:** (A) Mapa temático da razão de mortalidade materna; (B) Mapa temático da razão de mortalidade materna suavizada; (C) *Box Map* da razão de mortalidade materna; (D) *Moran Map* da razão de mortalidade materna, Pernambuco, Brasil, 2020–2022 (pandêmico)

*Fonte: SIM/GVEV/DGIE/SEVSAP/SES-PE.*

*Dados coletados em 4 de dezembro de 2023*

## DISCUSSÃO

No período do estudo, a maior parte dos óbitos ocorreu no período pandêmico, com o ano de 2020 registrando a maior RMM da série histórica. Houve predomínio de mortes entre as mulheres negras, com mais de oito anos de estudo e idades entre 10 e 34 anos. Em geral, eram multíparas com histórico de parto transvaginal. A maioria realizou o pré-natal com no mínimo seis consultas. Os óbitos se concentraram no puerpério e com predomínio de ocorrência nos serviços hospitalares. A principal causa de morte, que antes eram as hipertensões e hemorragias, passou a ser a COVID-19. Para a análise espacial, no período anterior à pandemia, observaram-se 19 municípios prioritários. Destes, 17 formam cinco clusters e dois apresentam-se de forma isolada. Durante o período pandêmico, houve redução nesse número, com o *Moran Map* identificando nove municípios prioritários. Porém, as principais áreas prioritárias de intervenção, nos períodos pré-pandêmico e pandêmico, concentram-se no Agreste do estado.

Nesta pesquisa, houve incremento na RMM em Pernambuco. A pesquisa exploratória conduzida no Brasil objetivou analisar os efeitos da pandemia pela COVID-19 na mortalidade materna. Como resultado, foi identificado o excesso de mortalidade durante a pandemia, estando relacionado a uma multicausalidade com articulação para além dos aspectos biológicos, envolvendo aspectos políticos, como o funcionamento e a estrutura do Sistema Único de Saúde e sua capacidade de gerir a crise sanitária(19). A pandemia introduziu barreiras adicionais à assistência na saúde obstétrica, o que afeta diretamente a mortalidade materna. A ocorrência do óbito por causas obstétricas é influenciada pela qualidade da assistência, que inclui o acesso aos serviços, à disponibilidade de recursos essenciais e à adequação das práticas em saúde durante o período gravídico-puerperal(20). A desestruturação dos serviços de saúde causada pela pandemia afetou, inclusive, os serviços de atenção primária, o que resultou em uma redução das consultas de pré-natal(21). Logo, doenças como pré-eclâmpsia/eclâmpsia, infecções urinárias, hemorragias, entre outras que deveriam ser diagnosticadas e tratadas no pré-natal, evoluíam com desfechos desfavoráveis(22). Dessa maneira, é preciso prioridade na recuperação do sistema de saúde nos territórios brasileiros, sobretudo no cenário pós-pandêmico(23). Em Pernambuco, faz-se necessário identificar territórios prioritários e atuar, com urgência, sobre estes a fim de minimizar os impactos da pandemia e retomar o projeto de redução da mortalidade materna.

Na análise espacial, em ambos os períodos, observou-se a identificação de municípios prioritários. Essas áreas possuem maior risco para ocorrência de mortalidade materna. Em semelhança, estudo conduzido no estado de Alagoas com dados dos óbitos por causas obstétricas entre os anos de 1996 a 2016, após análise espacial por meio da utilização do Índice de Moran, identificou regiões com maior risco para ocorrência de morte materna(24). Dessa maneira, a identificação de áreas prioritárias pode auxiliar no planejamento em saúde, monitoramento e direcionamento equitativo de intervenções e recursos em saúde que são, por vezes, insuficientes e escassos(25).

Os *clusters* identificados na pesquisa são, prioritariamente, formados por municípios do Agreste do estado. De acordo com dados do Índice de Desenvolvimento Humano Municipal (IDH-M), cerca de 77,5% dos municípios do Agreste de Pernambuco possuem um IDH-M classificado como baixo(26). O IDH-M utiliza dados sobre saúde, educação e renda per capita. Com isso, possibilita mensurar o nível de desenvolvimento da população em diferentes regiões(27). Uma pesquisa realizada em Pernambuco, entre os anos de 2010 e 2020, avaliou a autocorrelação espacial bivariada entre a mortalidade materna e o IDH-M por meio da estatística espacial de Moran. Como resultado, percebeu-se que os municípios com maior risco para óbito materno possuíam baixo desenvolvimento humano e pouco capital social estrutural(28).

Segundo dados do Acompanhamento Mensal da Taxa Estimada da População Pernambucana em Situação de Extrema Pobreza inseridas no Cadastro Único em relação à população estimada pelo IBGE, Pernambuco apresentou, em agosto de 2023, cerca de 37,8% da população em situação de extrema pobreza. No Agreste, 95,8% dos municípios apresentam percentuais superiores à média estadual e aproximadamente 67,6% dos municípios possuem mais de 50% de sua população residente em situação de extrema pobreza(26). Barreiras financeiras podem afetar negativamente a saúde materna em diferentes regiões, uma vez que promovem, entre outros, dificuldades de acesso a cuidados médicos regulares e emergenciais(29).

Um estudo realizado no Brasil, com dados sobre registros de 23.823 casos de COVID-19 e 1991 óbitos entre mulheres grávidas e puérperas, utilizou o Moran bivariado para avaliar a correlação espacial dos casos e óbitos de gestantes e puérperas por COVID-19 com dados socioeconômicos e de vulnerabilidade social. Como resultado, os municípios menores e aqueles com maior grau de vulnerabilidade apresentaram as mais elevadas razões de mortalidade materna(30).

Comumente, serviços de saúde interiorizados não estão adequadamente preparados para promover a assistência necessária a gestantes e puérperas. Isso pode incluir falta de recursos humanos qualificados, estruturas defasadas e baixas taxas de ocupação, o que compromete a qualidade do atendimento. Devido à ineficácia na prestação de assistência local, muitas vezes as gestantes precisam ser transferidas para unidades de saúde melhor equipadas(31). As dificuldades de transporte bem como a distância até os serviços de referência também representam um desafio(32). Regiões com maior desigualdade na qualidade e quantidade dos serviços de saúde potencializam os desfechos obstétricos negativos(33).

Estudo exploratório realizado no Brasil objetivou analisar o processo de regionalização em saúde no Brasil sob a ótica dos gestores municipais. Como resultado, observou-se que o acesso aos serviços de saúde, sobretudo de maior recurso tecnológico, é difícil e centralizado, o que exige que o usuário percorra grandes distâncias para conseguir atendimento(34). Outra pesquisa realizada no Agreste do estado de Pernambuco, com o objetivo de avaliar o acesso aos serviços de saúde, utilizou dados geográficos referentes à distância percorrida e ao tempo de deslocamento entre o município de residência e a ocorrência do parto. Como resultado, observou-se que 17,1% das mulheres que tiveram seu parto classificado como risco habitual e 49,1% classificados com alto risco precisaram se deslocar mais de 120 km até o serviço de saúde de referência(35).

Problemas estruturais, como as falhas no processo de regionalização e descentralização dos serviços de saúde, bem como nos sistemas de regulação, impactam negativamente no risco de desfechos obstétricos desfavoráveis. Portanto, a organização das redes de atenção à saúde no território, os sistemas de regulação integralmente implantados e um planejamento regional qualificado são determinantes para facilitar ou dificultar o acesso(31,32).

A combinação desses fatores exprime a complexidade dos desafios enfrentados pelos sistemas de saúde em áreas periféricas, em especial nos contextos onde os recursos em saúde são insuficientes e inadequados para lidar com emergências obstétricas. O que pode contribuir para o aumento da mortalidade materna nessas regiões.

Esta pesquisa aponta para um maior número de óbitos maternos no período pandêmico. Se não for tratado com prioridade, esse cenário poderá resultar em maiores dificuldades para a redução da mortalidade materna em Pernambuco. O compromisso brasileiro em reduzir a mortalidade por causa obstétrica para 30 óbitos maternos a cada 100.000 nascidos vivos se tornará mais um compromisso a ser postergado. Portanto, é urgente a necessidade de compreender os efeitos diretos e indiretos da pandemia pela COVID-19 a curto e longo prazos nos desfechos obstétricos.

Alcançar a meta proposta pelo ODS representa um desafio que precisa ser prioridade de gestão. É importante a garantia da continuidade dos serviços obstétricos para além da pandemia como estratégia fundamental de reestruturação e mitigação dos impactos negativos na saúde materna e infantil. Isso inclui políticas públicas que assegurem a continuidade do pré-natal, e outros cuidados essenciais, especialmente para territórios vulneráveis. Outro fator importante é a atuação dos gestores em saúde na descentralização e qualificação dos serviços de saúde nos locais periféricos. Essas medidas se traduzem em oportunidade para reduzir mortes evitáveis durante a gestação, o parto e o puerpério.

### Limitações do estudo

Como limitação do estudo, por se tratar de análise utilizando dados secundários, o preenchimento inadequado das declarações de óbito, especificamente sobre as causas de morte e o momento do óbito no período gravídico-puerperal, produz sub-registros das mortes maternas. O erro na codificação e/ou os erros na digitação da declaração de óbito no sistema contribuem na subnotificação e afetam as estimativas do indicador. Soma-se a isso que, para o ano de 2022, o dado é preliminar. No entanto, os dados dos Sistemas de Informações em Saúde em Pernambuco têm apresentado qualidade satisfatória relacionada aos eventos vitais e reafirmam a utilização desses sistemas como instrumentos confiáveis de análise da situação de saúde(36). Além disso, por se tratar de um estudo ecológico, é vulnerável à falácia ecológica. Dessa maneira, seus resultados não podem ser inferidos a nível individual. Por se tratar de um estudo descritivo, não possui capacidade de inferir relação de causa-efeito.

### Contribuições para as áreas de Enfermagem, Saúde e Políticas Públicas

A Enfermagem é fundamental para a atuação dos serviços de atenção primária à saúde, como por exemplo, as consultas de pré-natal. Esse nível de atenção possui a capacidade de resolução de cerca de 80% dos problemas de saúde. O óbito materno ocorre, em maior parte, por falhas de acesso e assistência às ações e aos serviços de saúde básicos e regulares. Os resultados do estudo evidenciam áreas prioritárias de intervenção que podem ser de interesse à gestão pública, tanto para a requalificação quanto para a descentralização dos serviços de saúde no estado.

## CONCLUSÕES

Os óbitos maternos apresentaram maior ocorrência no período pandêmico com alteração das áreas prioritárias entre os períodos. Apesar disso, as áreas prioritárias, tanto no período anterior à pandemia quanto durante o período pandêmico, concentram-se no Agreste Pernambucano.

A identificação geográfica de áreas prioritárias para ocorrência de óbitos maternos representa uma oportunidade de atuação local. É possível, com políticas e investimentos em saúde, reduzir a ocorrência de óbitos maternos em regiões de maior risco. Com isso, é possível que Pernambuco, com priorização, foco e comprometimento, alcance a meta estabelecida pelos ODS.

## Data Availability

Os dados de pesquisa só estão disponíveis mediante solicitação.
